# PET/MR for therapy response evaluation in malignant lymphoma: initial experience

**DOI:** 10.1007/s10334-012-0342-7

**Published:** 2012-09-16

**Authors:** Ivan Platzek, Bettina Beuthien-Baumann, Jens Langner, Manuel Popp, Georg Schramm, Rainer Ordemann, Michael Laniado, Jörg Kotzerke, Jörg van den Hoff

**Affiliations:** 1 Department of Radiology, Dresden University Hospital, Fetscherstr. 74, 01307 Dresden, Germany; 2 Department of Nuclear Medicine, Dresden University Hospital, Dresden, Germany; 3 Institute of Radiopharmacy, Helmholtz-Zentrum Dresden-Rossendorf, Dresden, Germany; 4 Medical Clinic and Polyclinic I, Dresden University Hospital, Dresden, Germany

**Keywords:** PET, MRI, Lymphoma

## Abstract

**Object:**

To evaluate the feasibility of positron emission tomography/magnetic resonance imaging (PET/MR) with ^18^fluoro-2-deoxyglucose (FDG) for therapy response evaluation of malignant lymphoma.

**Materials and methods:**

Nine patients with malignant lymphoma who underwent FDG-PET/MR before and after chemotherapy were included in this retrospective study. Average time between the two scans was 70 days. The scans were evaluated independently by two nuclear medicine physicians. The Ann Arbor classification was used to describe lymphoma stage. Furthermore, the readers also rated PET image quality using a five point scale. Weighted kappa (κ) was used to calculate interrater agreement.

**Results:**

The initial scan showed foci of increased FDG uptake in all patients, with Ann Arbor stage varying between I and IV. In the follow-up examination, all but one patient showed complete response to chemotherapy. PET image quality was rated as very good or excellent for all scans. Interrater agreement was excellent regarding Ann Arbor stage (κ = 0.97) and good regarding image quality (κ = 0.41).

**Conclusion:**

PET/MR shows promising initial results for therapy response evaluation in lymphoma patients.

## Introduction

Lymphomas are a diverse group of malignant diseases of the lymphocytes [1]. Lymphoma therapy has greatly improved in the last decades, and a large percentage of patients with lymphoma can now be cured.

Imaging has an important role in the initial staging, therapy response evaluation, and follow-up in lymphoma [2]. The aim of imaging in lymphoma patients is to identify lymph node groups affected by the disease and the eventual involvement of extralymphatic organs. The evaluation of morphology is often not sufficient for lymphoma staging, as malignant cells may be present in lymph nodes which are not enlarged or in residual masses often seen after therapy. In contrast to computed tomography (CT) and magnetic resonance imaging (MR), which focus on morphology, positron emission tomography (PET) allows for the evaluation of tissue metabolism. Due to the increased glucose metabolism of most lymphoma subtypes, PET with FDG (^18^fluoro-2-deoxyglucose) has become an established modality for lymphoma staging [2].

The impact of FDG-PET on lymphoma staging is reflected in recent recommendations of the International Harmonization Project. To facilitate the interpretation of post-therapy-PET, a baseline FDG-PET scan in patients with routinely FDG-avid lymphoma is encouraged, followed by the post-therapy scan 6–8 weeks after completion of chemotherapy [3]. Therapy monitoring in lymphoma patients helps identify non-responders, who may benefit from more aggressive therapy (salvage therapy) [4].

In the last decade, standalone PET scanners have been mostly replaced by PET/CT systems [5]. Thus PET is mostly performed as a part of a PET/CT examination, which also provides anatomic information thanks to the CT scan [6]. The recently introduced whole-body PET/MR systems [7] now offer the opportunity to combine PET with MRI instead. Prospectively, the functional imaging capabilities of MR, which are largely complementary to those offered by PET, might turn out to be especially relevant for combined PET/MR imaging. PET/MR also offers a superior soft tissue contrast and reduced overall radiation exposure in comparison to PET/CT. Thus PET/MR is a promising alternative to PET/CT in lymphoma staging and therapy monitoring.

The aim of this pilot study was to evaluate the feasibility of FDG-PET/MR for response evaluation of malignant lymphoma.

## Materials and methods

Nine patients (five men, four women, average age 31 years) with malignant lymphoma were included in this retrospective study. Lymphoma types included Hodgkin’s disease (*n* = 6), anaplastic large-cell lymphoma (*n* = 1), peripheral T cell lymphoma not otherwise specified (*n* = 1) and diffuse large B cell lymphoma (*n* = 1). Patient data are summarized in Table 1. All patients had undergone FDG-PET/MR twice, once for staging and once for response assessment. In four patients, the initial PET/MR scan was performed for staging of a newly diagnosed lymphoma, while the remaining five patients had lymphoma recurrence. Average time between the two scans was 70 days (41–164 days). In the interval between the two examinations all patients received chemotherapy and one patient also received autologous stem-cell transplantation.

**Table 1:** Patient information overview

Patient no.	Age	Sex	Lymphoma type	Uptake time 1. exam (min)	Injected FDG dose 1. exam (MBq)	Uptake time 2. exam (min)	Injected FDG dose 2. exam (MBq)	Additional CT scans available
1	28	f	Hodgkin’s disease	207	236	156	264	CT
2	34	f	Diffuse large B cell lymphoma	66	269	62	275	No
3	19	m	Hodgkin’s disease	98	351	99	280	No
4	21	m	Hodgkin’s disease	89	300	68	309	CT
5	64	m	Peripheral T cell lymphoma not otherwise specified	71	338	76	296	No
6	28	m	Anaplastic large-cell lymphoma	186	373	83	354	No
7	44	m	Hodgkin’s disease	84	206	74	223	No
8	15	f	Hodgkin’s disease	55	183	60	211	CT
9	22	f	Hodgkin’s disease	83	260	85	253	No

### PET/MR

The patients were instructed to restrain from food intake for at least 6 h before FDG-injection, while fluid intake (water or non-sugar added tea) was encouraged. FDG was administered intravenously 95 min (55–207 min) prior to the PET scan (183–373 MBq FDG, 277 MBq on average). Care was taken to adhere to comparable timing between FDG-injection and start of scan in both imaging sessions, with is routinely started 60–70 min p.i. In five out of eighteen scans, the time between the tracer injection and the beginning of the scan was shorter than 70 min. Substantially longer uptake time in two individual patients resulted from prior scanning on a dedicated PET system. This approach was taken for comparison reasons, since both patients had been scanned before on the dedicated PET system.

**Fig. 1:**
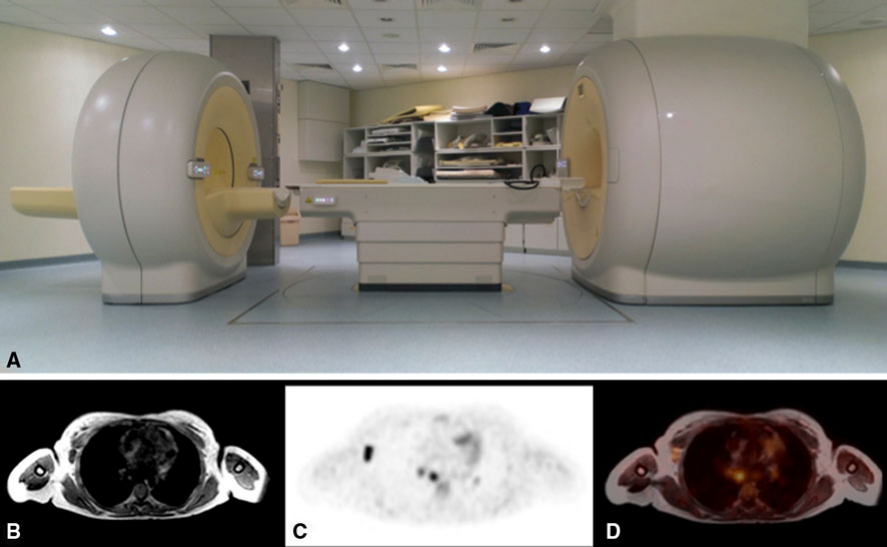
**a** Whole-body PET/MR system; **b** attenuation MR, a low-resolution gradient echo scan; **c** FDG-PET scan; **d** fused PET/MR image

PET/MR of all patients was performed with the Ingenuity TF PET/MR, (Philips Medical Systems, Best, Netherlands) shown in Fig. 1. The system is equipped with a 3T main magnet. The PET component of the system features time-of-flight technology, an axial field of view of 18, 9 cm overlap between bed positions and a reconstructed isotropic spatial resolution of ≈5.5 mm. The scintillation detectors of the system consist of LYSO scintillation crystals optically coupled to photomultiplier tubes [7].

With this system, PET and MR data are acquired sequentially, in analogy to PET/CT systems. The PET and MR gantry are connected with a rotating patient table. The distance between the centers of the PET and MR tunnels is 4.2 m. The purpose of this design is to minimize the influence of the magnetic field on the photomultiplier tubes used in the scintillation detectors. The magnetic flux is further reduced by laminated steel shielding around the scintillation detectors.

A PET/MR exam consists of a fast initial low-resolution nondiagnostic “attenuation MR scan” (atMR) followed by a PET scan, and may also include diagnostic MR, depending on the indication.

An atMR is a gradient echo scan, from which the attenuation map required for the PET reconstruction is generated. In short a MR-based tissue type segmentation and classification is performed, followed by an assignment of the known linear attenuation coefficients of the individual tissue types to the respective segments. The resulting attenuation map is then used for PET attenuation correction [8]. The atMR has to cover the whole area to be depicted by PET and is acquired with the integrated body coil. As the field of—view of the MR system has an axial extension of 50 cm, a multistage technique is used to achieve sufficient coverage, in analogy to PET. In the current study, atMR and the subsequent PET scan covered the patient from the skull base to mid-thigh. Acquisition parameters for the attenuation MR scan are summarized in Table 2.

**Table 2:** Sequence parameters of the attenuation MR scan (atMR)

TR (ms)	TE (ms)	Slice thickness (mm)	Stack thickness (mm)	Number of stacks	FoV (mm)	Acquisition time (min)
2.3	4.1	6.0	250	5	430 × 514	3:30

*TR* time to repeat, *TE* time to echo, *FoV* field of view

Ten to eleven bed positions were necessary to achieve PET coverage from the skull base to mid-thigh. Emission time was 2 min for each bed position, and the total PET scan time was 20–22 min.

The patients were examined in the supine position, with the arms down at sides. The position of the patient on the scanner table remains unchanged during the whole exam in order to maintain accurate coregistration of both imaging modalities.

In seven out of nine patients, PET/MR consisted of an attenuation MR scan and a PET scan, while in two patients both the initial exam and the follow-up exam included an additional diffusion-weighted MR scan (*DWIBS* diffusion weighted imaging with background suppression). The diffusion-weighted scan covered the neck, thorax, abdomen and pelvis and was acquired using the integrated body coil. In analogy to PET, a multistation technique is used to acquire a diffusion weighted dataset with sufficient coverage. Acquisition time for each set of diffusion-weighted images (i.e. stack) was 3:38 min. Seven stacks were necessary to achieve sufficient coverage, resulting in 24 min additional imaging time.

Total imaging time was 24–26 min without DWIBS (depending on the number of bed positions) and 48–50 min with DWIBS.

### Additional imaging studies available

Three patients had additional CT scans performed before and after chemotherapy on a 16-slice scanner (Somatom Sensation 16, Erlangen, Germany). Five out of six CT scans were acquired after intravenous contrast media injection. In each case 120 mm contrast medium was administered intravenously with a flow of 3 ml/s (Ultravist 370; Bayer Schering Pharma, Berlin, Germany), followed by a saline bolus chaser (40 ml). The delay after contrast media injection was 55 s.

In one CT scan no contrast medium was used.

Images were acquired with a tube voltage of 120 kV and a tube current of 120 mAs. The slice thickness was 3 mm.

In all three patients, the time interval between PET/MR and CT was under 16 days.

### Image analysis

The PET scans were evaluated independently by two nuclear medicine physicians who were blinded to other imaging tests. In addition, the attenuation maps, which were calculated from the atMR for attenuation of the PET data, were reviewed for possible artifacts. Both readers used the ROVER^®^ software package (ABX advanced biochemical compounds, Radeberg, Germany) for viewing PET images. It allows for the viewing of PET data in arbitrary slice orientation and also the calculation and viewing of MIP (maximum intensity projection) PET images. The Ann Arbor staging system [9] was used to describe the findings. PET datasets were assessed visually for artifacts and image contrast. Overall PET image quality was rated using a scale between 1 and 5 (1 = poor, 2 = fair, 3 = good, 4 = very good, 5 = excellent). Weighted kappa was used as a measure of interobserver reliability of lymphoma staging and image quality assessment. Statistical analysis was performed using MedCalc 12.0 (MedCalc Software bvba, Mariakerke, Belgium). A *p* value ≤0.05 was considered statistically significant.

The atMR and the DWIBS were evaluated by a radiologist. The concordance between PET dataset and atMR was evaluated together by a nuclear physician and a radiologist. In cases with additional CT exams, these were evaluated by a radiologist blinded for the PET/MR data.

## Results

### PET

In the initial scan, one patient had Ann Arbor stage I disease, two patients had stage II disease, three patients had stage III disease and three patients had stage IV disease. In total, 130 lesions were detected using PET/MR, including 128 lymph nodes, one pulmonary lesion, and one liver lesion.

**Fig. 2:**
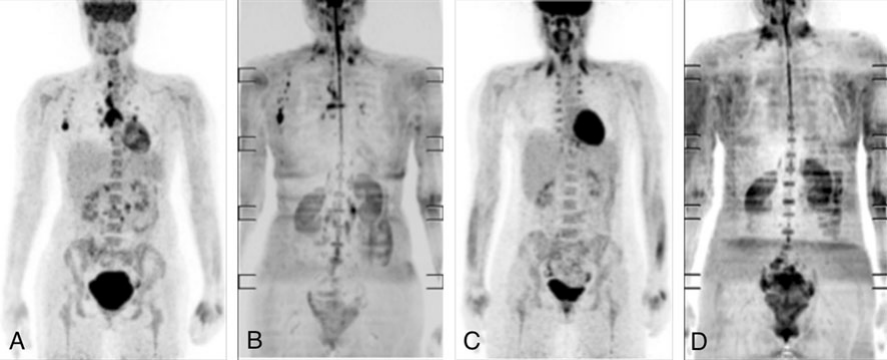
FDG-PET/MR in a patient with Hodgkin’s disease before and after chemotherapy. **a** PET MIP image showing enlarged lymph nodes with increased FDG uptake in the mediastinum and the right axilla. **b** Inverted MIP of diffusion weighted MR images acquired during the same exam, with the lymphoma mentioned above clearly recognizable. **c** PET MIP after chemotherapy, showing complete response. **d** Corresponding inverted DWIBS MIP. Non-specific symmetric FDG-accumulation in brown fat tissue in the lower cervical and supraclavicular region on both PET scans (more pronounced on the second PET with additional paravertebral symmetric FDG uptake) should not be mistaken as lymphoma tissue. Signal decrease in the lower cervical region on the inverted diffusion weighted images is caused by inhomogenous fat suppression (DWIBS Images are acquired with the integrated body coil of the MR system)

On the follow-up scan, eight out of nine patients showed complete remission, as shown in Fig. 2, while one patient had residual disease after therapy (stage III).

Both readers had identical results regarding Ann Arbor stage in 17 out of 18 scans, and differed in one case, resulting in a weighted kappa value of 0.97. The discrepancy resulted from a single infradiaphragmal lymph node with increased FDG uptake that was missed by one of the readers.

All PET datasets were found to have very good or excellent image quality. Reader 1 rated 11 scans as excellent and seven as very good, while reader 2 rated 16 scans as excellent and two as very good. For interrater agreement regarding image quality we calculated a weighted kappa of 0.41.

### Attenuation map

**Fig. 3:**
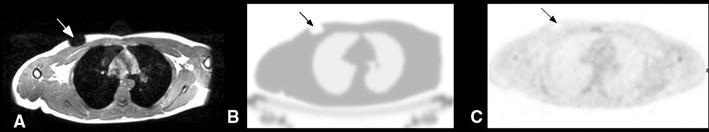
Artifacts caused by a port system impacted on the right side of the patients chest. **a** Susceptibility artifacts on the attenuation MR scan (*arrow*). **b** Corresponding defect on the MRMap, a template calculated from the attenuation scan and used for attenuation correction. **c** On the PET scan, the port is recognizable as an area without visible FDG uptake (*arrow*). Image quality in the surrounding area does not appear to be degraded by the metal artifacts

No major artifact occurred on the attenuation maps, and air filled organs like the lungs and trachea were identified correctly by the segmentation algorithm. In three scans an infraclavicular port system was identified, causing a focal localized defect (Fig. 3), but no further artifacts on the PET images.

### atMR

In 11/18 atMR scans pulsation artifacts from the aortic arch were visible mainly in the right upper lung. This artifact did not interfere with the overall evaluation of the MR data. The pulsation artifacts were not carried forward into the attenuation maps and did not alter the attenuation correction of the PET data.

### Coregistration of atMR and PET

The image fusion of atMR and PET showed a small misalignment at the level of the diaphragm in 2/18 investigations. The misalignment in these two studies can be attributed to different breathing depth during the atMR and the PET acquisition. The image interpretation was not impaired. No relevant misalignment between both scans were detected.

### DWIBS

DWIBS of both imaging time points were only available for two patients. In both patients lesions with restricted diffusion were visible on the initial scan before treatment. In these two patients, FDG-PET/MR detected 28 lymph nodes with increased FDG uptake (21 and 7 lymph nodes, respectively). Lesion locations included the mediastinum, the axilla and supraclavicular fossa. In contrast, 24 lymph nodes with restricted diffusion were detected using DWIBS. The four lymph nodes which were suspected for lymphoma involvement on the PET scan but were classified as normal by visual evaluation of the DWIBS images were located in the left axilla.

After treatment, both patient were classified as complete response with both PET and DWIBS.

### Comparison with CT

Three patients had additional CT scans performed before and after chemotherapy.

Nine lesions were identified in those patients before therapy in total, including cervical lymph nodes (*n* = 4), axilary lymph nodes (*n* = 1), mediastinal lymph nodes (*n* = 1), retroperitoneal lymph nodes (*n* = 1), one liver lesion and one pulmonary lesion.

**Fig. 4:**
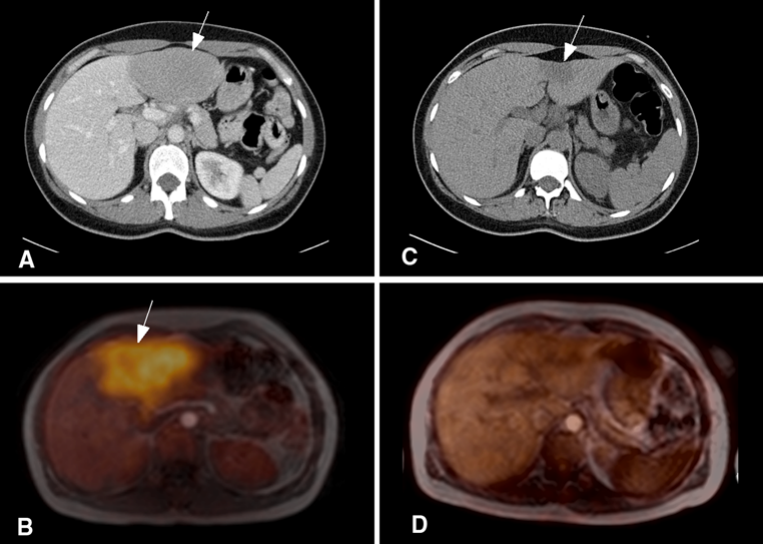
FDG-PET/MR and corresponding CT images of a patient with diffuse large B cell lymphoma. **a** Contrast enhanced CT scan showing a hepatic lymphoma manifestation before chemotherapy. **b** Fused FDG-PET/MR before chemotherapy. **c** Nonenhanced CT scan after chemotherapy showing a residual mass (*arrow*). **d** Corresponding PET/MR after chemotherapy showing no pathologic FDG uptake

All lesions were identified on the initial PET/MR and CT scans. Both modalities yielded concordant results for initial Ann Arbor stage (one patient with stage II disease, one with stage III and one with stage IV). After chemotherapy, comparison between PET/MR and the CT scans showed concordant findings for six lesions and discordant findings for three lesions. The discordant cases included a residual liver mass (Fig. 4), a residual mediastinal mass and a remaining enlarged axillary lymph node, which were all FDG-negative. Thus staging results after chemotherapy differed between CT and PET/MR for two patients, who showed no metabolic signs of disease on the PET scan, but were suspected of having residual disease using the CT scan. The third patient had residual disease after chemotherapy, which was identified by both CT and PET/MR.

## Discussion

The current study shows that FDG-PET/MR can be used for therapy response evaluation in lymphoma and provides PET datasets with good image quality.

From the technical point of view, pulsation artifacts in atMR as well as small metal artifacts in atMR and the attenuation map did not interfere with the high quality of the PET scans. The spatial correspondence between atMR and PET was good, and no relevant major misalignment between both scans was observed.

The excellent interobserver agreement regarding Ann Arbor stage implies that FDG-PET/MR is a reliable imaging method. While interobserver agreement for image quality was not that high, it should be noted that all scans were rated as either very good or excellent. Discrepancies between the readers may be influenced by the fact that in contrast to Ann Arbor staging, there are no clear-cut criteria to categorize image quality.

Follow-up PET scans in lymphoma are performed to evaluate changes in FDG uptake and thus anatomic images are of less importance in such scans. In comparison to PET/CT the radiation dose is reduced, as MR does not use ionizing radiation. As lymphoma often occur in young patients and repeated PET scans are needed for response assessment, the reduced radiation exposure is an important advantage of PET/MR. For example, Nievelstein et al. calculated a cumulative effective dosis of 97 mSv 2.5 years after diagnosis in adults with non-Hodgkin’s lymphoma being monitored with PET/CT [10]. Effective dose for a single whole-body CT scan was 13.3 mSv and for FDG-PET 4.2 mSv [10]. Thus the cumulative radiation dose could be reduced significantly by replacing the CT scan with an MR scan.

Without diagnostic MR, no significant difference of total acquisition time is expected between sequential PET/MR systems, as described above, and PET/MR systems with simultaneous acquisition, which has also become available [11]. Including diagnostic MRI like DWIBS prolongs total imaging time within a clinical well acceptable range.

Furthermore, PET/MR is a promising research tool. For instance, in the last few years there has been increasing interest in extracranial applications of diffusion-weighted MRI, including lymphoma staging with DWIBS [12, 13]. While whole-body diffusion weighted imaging of lymphoma is technically feasible, its clinical role still has to be defined. PET/MR offers the unique opportunity to evaluate the differential or integral impact of DWIBS and FDG-PET in staging and restaging of lymphoma.

No PET/CT studies were available for comparison with PET/MR. In patients who underwent additional CT scans, the comparison with PET/MR showed differing results for lymphoma stage, which are caused by the inability of CT to evaluate tissue metabolism.

Among the shortcomings of the current study are the small number of patients and the different types of lymphoma included. However, the focus of the study was on feasibility and image quality as the fundamental role of FDG-PET for lymphoma imaging has already been established before the introduction of PET/MR. Also, long-term follow-up is not yet available for these patients. Thus further studies are necessary to evaluate the prognostic value of FDG-PET/MR-focusing on FDG-PET and the combination with diagnostic MRI in lymphoma.

## Conclusion

FDG-PET/MR is a promising method for monitoring lymphoma therapy and allows for the acquisition of high quality PET datasets in a reasonable timeframe.
